# Effects of pulmonary-based Qigong exercise in stable patients with chronic obstructive pulmonary disease: a randomized controlled trial

**DOI:** 10.1186/s12906-023-04238-8

**Published:** 2023-11-20

**Authors:** Linhong Jiang, Peijun Li, Jiacheng Shi, Yidie Bao, Zhenwei Wang, Weibing Wu, Xiaodan Liu

**Affiliations:** 1 School of Rehabilitation Science, Shanghai University of Traditional Chinese Medicine, Shanghai, 201203 P.R. China; 2 Department of Respiratory Medicine, Shanghai University of Traditional Chinese Medicine Yueyang Hospital of Integrated Traditional Chinese Medicine and Western Medicine, Shanghai, 200437 P.R. China; 3 Department of Sports Rehabilitation, Shanghai University of Sport, Shanghai, 200438 P.R. China; 4 Engineering Research Center of Traditional Chinese Medicine Intelligent Rehabilitation, Ministry of Education, Shanghai, 201203 P.R. China; 5 Institute of Rehabilitation Medicine, Shanghai Academy of Traditional Chinese Medicine, Shanghai, 201203 P.R. China

**Keywords:** Traditional Chinese exercise, Chronic obstructive pulmonary disease, Rehabilitation

## Abstract

**Background:**

Physical exercise training is the central component of pulmonary rehabilitation. This study aimed to further investigate the rehabilitative effects of pulmonary-based Qigong exercise (PQE) in stable patients with chronic obstructive pulmonary disease (COPD).

**Methods:**

In this randomized, assessor-blinded clinical trial, 44 participants with stable COPD were randomly assigned to 2 groups in a 1:1 ratio. Participants in the control group received usual care for 3 months. Participants in the intervention group received usual care combined with PQE (60 min each time, 2 times per day, 7 days per week, for 3 months). The outcome included exercise capacity, lung function test, skeletal muscle strength, dyspnea, and quality of life were measured before and after intervention.

**Results:**

A total of 37 participants completed the trial. Compared to the control group, after 3 months of PQE, the mean change in exercise capacity, skeletal muscle strength, and quality of life were statistically significant (*P* < 0.05, for each), but no significant differences were observed in lung function (except for the forced expiratory volume in one second) and dyspnea (*P* > 0.05, for each).

**Conclusion:**

The findings of study suggest that the proposed program of 3 months of PQE intervention has significant improvement in exercise capacity, skeletal muscle strength, and quality of life of COPD-stable patients.

**Trial registration:**

This study was registered in the Chinese Clinical Trial Registry (Trial ID: ChiCTR-1800017405 on 28 July 2018; available at https://www.chictr.org.cn/showproj.html?proj=28343).

**Supplementary Information:**

The online version contains supplementary material available at 10.1186/s12906-023-04238-8.

## Introduction

Currently, chronic obstructive pulmonary disease (COPD) is a global health emergency that affects people from all countries, socioeconomic classes, and age groups, and it has become one of the top three causes of death worldwide [1]. COPD is a heterogeneous lung condition characterized by chronic respiratory symptoms due to abnormalities of the airways and/or alveoli that cause persistent, often progressive, airflow obstruction [2]. Importantly, COPD is a systemic disease that not only affects the local lungs, causing decreased lung functions and dyspnea, but also causes multisystemic implications and comorbidities including skeletal muscle function, which can lead to decreased exercise capacity and poor quality of life [3, 4].

Physical exercise training, as an active treatment recommended by the American Thoracic Society/European Respiratory Society with strong evidence, is the central component of pulmonary rehabilitation (PR) [5]. Traditional Chinese exercise (TCE) is an ancient Chinese system of gentle, self-healing physical exercise training designed to improve body function, and it is practiced in many Asian communities and has growing popularity in Western countries [6]. Previous meta-analyses have demonstrated that TCE (including Tai Chi, Qigong, Liu Zijue, Wu Qinxi, and Ba Duanjin) is beneficial for improving lung function, mobility or physical performance, and quality of life in patients with COPD [7, 8], and a recent randomized controlled trial reported that Qigong exercise and cycle ergometer exercise had similar rehabilitation effects on the improvement of the cardiopulmonary endurance and quality of life of COPD patients [9]. However, those trials have focused on a single type of TCE as the intervention with inconsistent clinical effects on COPD patients based on the exercise type, and some movements are physically draining and do not help the patients improve.

**Fig. 1:**
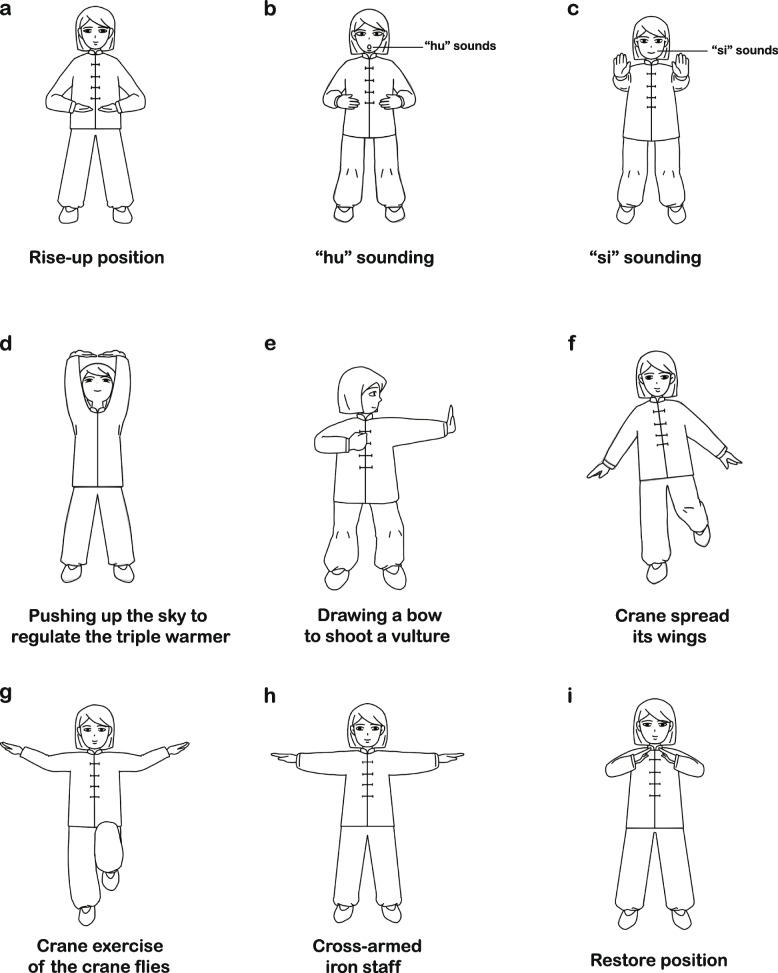
Main characteristics of pulmonary-based Qigong exercise. **a** Rise-up position. **b** “hu” sounding. **c** “si” sounding. **d** Pushing up the sky to regulate the triple warmer. **e** Drawing a bow to shoot a vulture. **f** Crane spread its wings. **g** Crane exercise of the crane flies. **h** Cross-armed iron staff. **i** Restore position

Compared with a single type of TCE, pulmonary-based Qigong exercise (PQE) is more targeted and practical for COPD-stable patients' functional recovery, which was developed by Liu et al. [10] based on the disease characteristics of COPD. The PQE, following the basic theory of traditional Chinese medicine, combines and reorganizes elements from Liu Zijue, Wu Qinxi, Ba Duanjin, and Yi Jinjing to compile a new intervention of prescribed pulmonary exercise for COPD rehabilitation. Basing on the traditional Chinese medicine theory, the vitality of the energy called Qi, and the vital energy flows through all of the organ systems and tissues of the body via channels called meridians and collaterals [7]. The main characteristics of PQE include the “hu” sounding (regulating the spleen to ensure the production of Qi) and “si” sounding (regulating the lung to ensure the Qi is dispersed and purged) in Liu Zijue, “pushing up the sky to regulate the triple warmer” (regulating Qi activity to dredge channels and collaterals) and “drawing a bow to shoot a vulture” (clearing the Lung Meridian of Hand-Taiyin to regulate the Qi of the lung) in Ba Duanjin, “crane spread its wings and crane exercise of the crane flies” (flowing the Qi of conception and governor vessels) in Wuqinxi, and “cross-armed iron staff” (regulating the flow of Qi and relieving de-pression in the chest) in Yi Jinjing. Overall, the aforementioned characteristics of PQE are beneficial to tonify lung Qi, and to dredge channels and collaterals of body, thereby enhancing the physical functions of COPD patients. In addition to regulating Qi, PQE achieves the effect of strengthening muscles and bones through mild to moderate aerobic activity and strength training of the core muscles, upper and lower limbs muscles, and respiratory muscles, which are important aspects of COPD management. The Fig. 1 and Additional file 1 shows the details of PQE training.

A previous study has found that PQE intervention can significantly improve exercise capacity and facilitate activities of daily living and social participation compared to conventional PR [11]. However, this study did not examine the effects of PQE intervention on skeletal muscle strength, lung function, and physical activity in COPD-stable patients. So, basing on prior research, the present trial further investigates the rehabilitative effects of PQE intervention on COPD-stable patients using multidimensional outcomes. We hypothesized that PQE intervention may improve lung function, exercise capacity, skeletal muscle function, dyspnea, and quality of life of COPD-stable patients.

## Methods

### Study design

This study was designed as a randomized, superiority, assessor-blinded clinical trial with two parallel groups of intervention and control. The study protocol was reviewed and approved by the Institutional Review Board of Shanghai University of Traditional Chinese Medicine Yueyang Hospital of Integrated Traditional Chinese Medicine and Western Medicine, Shanghai, China (2018–080), and was registered on the Chinese Clinical Trial Registry (ChiCTR-1800017405). All participants provided written consent. This clinical trial adheres to the CONSORT 2010 guidelines for reporting randomized trials [12].

### Participants

From June 2018 to October 2019, participants with stable COPD were recruited and screened in the Department of Respiratory, Shanghai University of Traditional Chinese Medicine Yueyang Hospital of Integrated Traditional Chinese Medicine and Western Medicine (Shanghai, China). Participants were mainly recruited through publishing recruitment announcements in posters, and WeChat promotions with the eligibility criteria of the trial.

The diagnosis of COPD was confirmed following the 2017 Global Initiative for Chronic Obstructive Lung Disease criteria [13]. Inclusion criteria are as follows: (1) Participants were diagnosed with moderate to very severe COPD (stages II-IV) [13] and they have been clinically stable in the 4 weeks before randomization; (2) No gender limitation, age 40 to 80 years; (3) Participants have not participated in any organized exercise training (at least twice a week) in the past 6 months. (4) Agreement of the participants to voluntarily participate in the trial and signed informed consent. Able to understand and implement rehabilitation training.

Exclusion criteria are as follows: (1) Unable to communicate or unable to follow commands; (2) Acute exacerbation that requires a change in pharmacological management or hospitalization; (3) Coexistence of other chronic respiratory disorders; (4) Skeletal muscle disease or other disease hampering assessment of muscle strength; (5) Current participation in any experimental trial.

### Sample size

Assessment of six minutes walking test (6MWT) as the primary outcome. According to the results of a similar study conducted previously [14], the sample size was based on detecting a minimum difference of 54 m in the 6MWT between the control group and the intervention group and used a baseline standard deviation of 57 m. In this study, a two-tailed test was chosen (α = 0.05), considering 80% power, using the statistical formula of mean difference. The smallest sample size was determined to be 38. However, considering the attrition rate of 15%, the sample size was increased to 44, with 22 participants included in each group.

### Randomization and blinding

All eligible participants were randomly assigned to two groups in a 1:1 ratio. Computerized random numbers were generated using the Statistical Package for the Social Sciences (SPSS) 26.0 statistical software. This work was conducted by an independent investigator and kept in a sealed, opaque envelope. When eligible participants were enrolled, the physiotherapists opened the envelopes in order of numbering on the envelopes to view the randomly assigned groups. The occupational therapist will give the participant the corresponding intervention. The trial is an exercise therapy intervention study, therefore, the therapist and participants will not be blinded by the assigned treatment. However, the outcome assessors, data collectors, and data analysts were unaware of the group assignments to maintain blinding.

## Interventions

### Control group

Eligible participants who were assigned to the control group received the usual care provided based on the 2017 Global Initiative for Chronic Obstructive Lung Disease guidelines [13], including prescribed medication, smoking cessation, and education. During the trial, participants did not receive any form of regular exercise intervention (> 2 times per week for > 60 min each), and the investigators conducted regular weekly telephone visits to ask participants for information about their medication, exercise, and daily activity for the week to ensure compliance with the trial. Participants were classified as compliant if the completion of responses to the telephone calls was no less than 85%.

### PQE group

The participants in the exercise group also accepted the usual care provided, which is identical to the control group. The difference between the two groups is that the exercise group conducted PQE intervention for 60 min each time, 2 times per day, 7 days per week, for 3 months. This exercise program is primarily based on participants themselves at home: On Sunday afternoons, ask participants to gather in the hospital to perform exercises under the supervision and instruction of a physiotherapist, and for the remaining 6 days to perform exercises at home. The exercise protocol consists of three actions, and the operation processes are as follows.

- **Action 1**: Participants are instructed to perform a warm-up exercise for 5–10 min that focused primarily on dynamic flexibility exercises of involved muscle groups and stretching muscles.
- **Action 2**: Participants are instructed to perform PQE intervention for 40 min. The exercise consists of nine characteristics: rise-up position, the “hu” and “si” sounds in Liu Zijue, “pushing up the sky to regulate the triple warmer” and “drawing a bow to shoot a vulture” in Ba Duanjin, “crane spread its wings and crane exercise of the crane flies” in Wuqinxi, and “cross-armed iron staff” in Yi Jinjing, restore position (Fig. 1). The detailed action plans are presented in Additional file 1.
- **Action 3**: Participants are instructed to perform a cool-down exercise for 5–10 min that focused primarily on stretching and relaxing their muscles.

To ensure participants' compliance with the exercise intervention, importantly, here are some important operation processes that should be performed before the formal training session: (1) Give training instructions 3 times within 2 weeks to participants; (2) Deliver the video of the prescribed pulmonary exercises and the exercise record brochure to all participants; (3) Ask participants to study the complete action and breathing requirements of each intervention; (4) Ask participants to record detailed information after each exercise session in the exercise record brochure, including details about exercise method, time, duration, intensity, and site, and submit the exercise record information of the week at each intensive training; (5) Ask participants to send videos of their daily home PQE training to the physiotherapist, and the physiotherapist gives the participants feedback based on the videos to ensure the effects of home exercises for patients; (6) Participants are instructed to take a break if they feel tired, labored, or have difficulty breathing during the exercise and to wait until the abnormal sensations disappeared before continuing to complete the exercise. All exercise actions are administered by a trained physiotherapist. Additionally, evaluate the exercise intensity using the Borg category-ratio 10 (Borg CR-10) and ask participants to maintain a level of dyspnea in the range of 4 to 6 [15]. In the hospital, the Borg CR-10 and heart rate monitor (Polar team 2) are both used to evaluate exercise intensity. Compliance was quantified by the completion of the exercise log by participants and attendance at the supervised training sessions in the hospital, with compliance defined as no less than 85% of exercise sessions completed.

### Outcomes

Outcome measures were performed by a certified, trained physiotherapist who is blinded to the group allocation of the participants. The assessments were administered at baseline and after completion of the intervention (3 months).

The primary outcome measure is a change in the distance on the 6MWT. The 6MWT is conducted following the American Thoracic Society guidelines. The participants are tested for the longest distance walked in 6 min over a flat 30 m linear distance, which is a submaximal exercise test used to quantify the functional exercise capacity in clinical populations [16].

The secondary outcome measures include lung function tests, skeletal muscle strength, dyspnea, and quality of life. The lung function test is performed by a dedicated medical staff in the lung function room using a lung function device (Masterscreen-PFT, Jaeger, Germany), which includes forced vital capacity (FVC), forced expiratory volume in one second (FEV1), percentage of predicted values of FEV1 (FEV1%pred), FVC% pred, and FEV1/FVC %. Skeletal muscle strength is assessed using the isokinetic muscle strength test (CON-TREX, Physiomed, Germany). The isokinetic muscle strength test can assess dynamic muscle strength, and the data output can reflect the functional status of specific muscles [17]. The tests are evaluated by trained staff following the relevant requirements for elbow and knee isokinetic strength tests given in the instrument guidelines, and the test indicators include peak torque (PT), PT to body weight ratio (PT/BW), total work (TW), and endurance ratio (ER). The symptoms of dyspnea are assessed using the Modified Medical Research Council (mMRC) Dyspnea Scale, a five-point scale (0–4) of the severity of dyspnea, with a higher score indicating a higher severity [18]. The quality of life is assessed using St. George's Respiratory Questionnaire (SGRQ). The SGRQ has a total of 50 questions that can be categorized into three items including symptoms, activities, and impacts. Scores range from 0 to 100, with higher scores indicating poorer quality of life [19].

### Statistical analysis

Data were analyzed using SPSS version 26.0 software (IBM Corporation, Armonk, USA). A normality test and variance homogeneity test were used to measure the continuous variables. The variables were described as means and standard deviations.

Inter-group comparison was performed using the analysis of covariance, with the baseline values as the covariates. Intra-group comparison was performed using the paired *t*-test. Categorical variables were tested with Chi-squared test or Fisher’s exact test, and the variables were described as frequencies (in percentage). Statistical significance was set at a *P* value less than 0.05.

## Results

### Participant characteristics

**Fig. 2:**
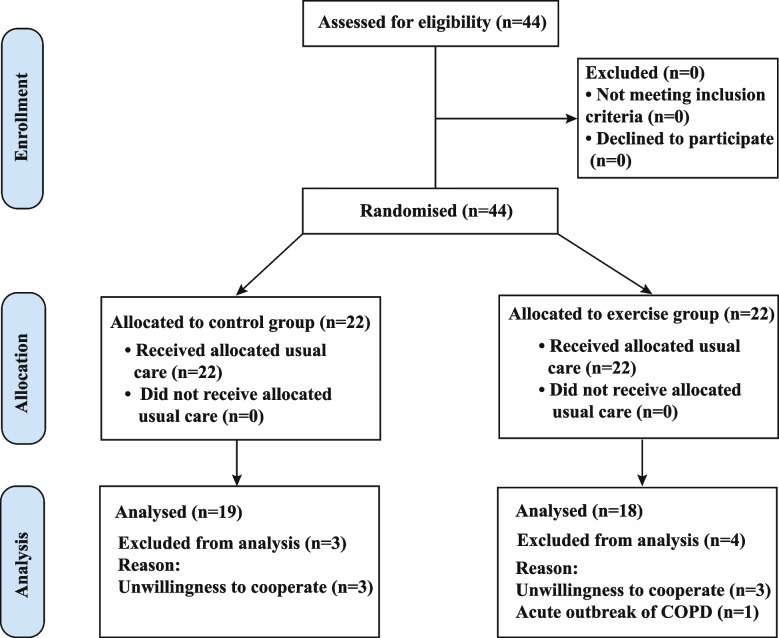
Flowchart of study participants

Thirty-seven participants completed the trial, with 19 participants and 18 participants in the control group and PQE group, respectively. Figure 2 shows the flow of participants from registration to the end of the trial. Seven participants were excluded from the study (3 in the control group and 4 in the PQE group) for the following reasons: unwillingness to continue the trial due to personal reasons (*n* = 6), and acute outbreak of COPD (*n* = 1). The characteristics of all participants are shown in Table 1, including age, gender, body mass index, year of COPD, COPD stage, and mMRC score. There was no statistically significant difference between the two groups in terms of participant characteristics (*P* > 0.05, for each; Table 1).

**Table 1:** Baseline characteristics of participants

Characteristics	Control group (*n* = 19)	PQE group (*n* = 18)	*P*-value
Age (years)	64.58 ± 9.06	66.11 ± 9.08	^†^ 0.611
Gender (male/female, n%)	14 (73.7%) /5 (26.3%)	15 (83.3%) /3 (16.7%)	^*^0.693
BMI, kg/m^2^	22.90 ± 3.71	25.22 ± 0.82	^†^ 0.058
Years of COPD	11.12 ± 4.66	10.28 ± 5.67	^†^ 0.640
COPD stage, n (%)			^*^ 0.834
Grade I	2 (11%)	1 (6%)	
Grade II	11 (58%)	10 (55%)	
Grade III	5 (26%)	7 (39%)	
Grade IV	1 (5%)	0 (0%)	
mMRC score, n (%)			^*^ 0.598
Grade 0	1 (5%)	2 (11%)	
Grade 1	3 (16%)	3 (17%)	
Grade 2	11 (58%)	12 (67%)	
Grade 3	4 (21%)	1 (5%)	
Grade 4	0 (0%)	0 (0%)	

*Abbreviations*: *BMI* Body mass index, *COPD* chronic obstructive pulmonary disease, *mMRC* modified Medical Research Council, *PQE* pulmonary-based Qigong exercise

-Values are presented as means and standard deviations or n (%). *P*-value based on: † Independent *t* test; * Fisher’s exact test

### Effect of PQE intervention on outcomes

#### Primary efficacy outcome

##### Exercise capacity

In Table 2, inter-group comparison revealed a significant difference in 6MWT between PQE group and control group (*P* < 0.001). Additionally, intra-group comparison showed a significant difference in PQE group (*P* = 0.005) and no significant difference in control group (*P* = 0.074), respectively.

**Table 2:** Comparison of outcomes in treatment groups before and after intervention

Outcome variables	Measurement period	Control group(*n* = 19)	PQE group(*n* = 18)	*P*^a^
Exercise capacity				
6MWT (m)	Before intervention	440.74 ± 82.00	501.26 ± 74.08	< 0.001
	After intervention	434.07 ± 83.12	535.78 ± 55.09	
	*P*^b^	0.074	0.005	
Lung function test				
FEV1 (L)	Before intervention	1.52 ± 0.57	1.63 ± 0.57	0.044
	After intervention	1.37 ± 0.46	1.66 ± 0.58	
	*P*^b^	0.091	0.717	
FVC (L)	Before intervention	2.52 ± 0.85	2.74 ± 0.72	0.071
	After intervention	2.37 ± 0.74	2.78 ± 0.64	
	*P*^b^	0.246	0.633	
FEV1% pred (%)	Before intervention	59.16 ± 18.15	59.18 ± 18.41	0.460
	After intervention	57.49 ± 16.88	59.57 ± 20.01	
	*P*^b^	0.326	0.876	
FVC% pred (%)	Before intervention	74.67 ± 19.68	82.33 ± 27.63	0.140
	After intervention	71.09 ± 20.02	83.56 ± 25.81	
	*P*^b^	0.326	0.584	
FEV1/FVC% (%)	Before intervention	60.00 ± 8.87	58.86 ± 10.42	0.901
	After intervention	57.47 ± 13.59	58.53 ± 11.00	
	*P*^b^	0.764	0.672	
Skeletal muscle strength				
Elbow isokinetic strength test				
PT(Nm)-Extensor muscle	Before intervention	36.83 ± 8.93	25.86 ± 9.03	0.415
	After intervention	36.70 ± 8.75	29.95 ± 8.52	
	*P*^b^	0.757	0.068	
PT(Nm)-Flexor muscle	Before intervention	37.72 ± 8.66	30.86 ± 8.44	0.226
	After intervention	37.43 ± 7.91	34.94 ± 8.51	
	*P*^b^	0.598	0.083	
PT/BW(Nm/kg)-Extensor muscle	Before intervention	0.59 ± 0.12	0.35 ± 0.13	0.919
	After intervention	0.58 ± 0.11	0.44 ± 0.12	
	*P*^b^	0.626	0.027	
PT/BW(Nm/kg)-Flexor muscle	Before intervention	0.60 ± 0.12	0.42 ± 0.11	0.584
	After intervention	0.60 ± 0.11	0.51 ± 0.14	
	*P*^b^	0.532	0.049	
TW(J)-Extensor muscle	Before intervention	374.17 ± 113.21	623.89 ± 120.37	0.281
	After intervention	385.44 ± 151.85	658.97 ± 132.12	
	*P*^b^	0.338	0.029	
TW(J)-Flexor muscle	Before intervention	367.19 ± 148.22	699.39 ± 169.09	0.237
	After intervention	360.53 ± 150.97	737.81 ± 181.89	
	*P*^b^	0.168	0.069	
ER-Extensor muscle	Before intervention	0.67 ± 0.12	0.83 ± 0.13	0.001
	After intervention	0.64 ± 0.16	1.06 ± 0.67	
	*P*^b^	0.347	0.264	
ER-Flexor muscle	Before intervention	0.69 ± 0.11	0.87 ± 0.10	0.081
	After intervention	0.69 ± 0.13	0.88 ± 0.07	
	*P*^b^	0.441	0.659	
Knee isokinetic strength test				
PT(Nm)-Extensor muscle	Before intervention	75.46 ± 23.45	74.56 ± 30.79	0.005
	After intervention	73.86 ± 22.39	79.92 ± 28.00	
	*P*^b^	0.178	0.023	
PT(Nm)-Flexor muscle	Before intervention	47.28 ± 19.38	45.18 ± 18.82	0.025
	After intervention	45.63 ± 18.46	49.16 ± 15.85	
	*P*^b^	0.108	0.090	
PT/BW(Nm/kg)-Extensor muscle	Before intervention	1.18 ± 0.28	0.96 ± 0.37	0.017
	After intervention	1.15 ± 0.27	1.12 ± 0.40	
	*P*^b^	0.172	0.019	
PT/BW(Nm/kg)-Flexor muscle	Before intervention	0.73 ± 0.23	0.59 ± 0.23	0.012
	After intervention	0.71 ± 0.23	0.72 ± 0.27	
	*P*^b^	0.118	0.021	
TW(J)-Extensor muscle	Before intervention	850.12 ± 351.37	1204.58 ± 482.78	0.003
	After intervention	836.87 ± 332.09	1284.01 ± 513.53	
	*P*^b^	0.381	0.003	
TW(J)-Flexor muscle	Before intervention	647.67 ± 237.43	805.01 ± 298.48	0.001
	After intervention	587.66 ± 214.76	836.46 ± 302.80	
	*P*^b^	0.016	0.016	
ER-Extensor muscle	Before intervention	0.63 ± 0.22	0.91 ± 0.16	0.376
	After intervention	0.61 ± 0.19	0.86 ± 0.14	
	*P*^b^	0.183	0.199	
ER-Flexor muscle	Before intervention	0.67 ± 0.13	0.85 ± 0.10	0.351
	After intervention	0.62 ± 0.14	0.80 ± 0.09	
	*P*^b^	0.001	0.141	
Quality of life				
SGRQ score				
Total	Before intervention	37.16 ± 15.02	36.18 ± 11.76	< 0.001
	After intervention	37.47 ± 17.82	23.29 ± 10.59	
	*P*^b^	0.833	< 0.001	
Symptom	Before intervention	52.16 ± 18.43	53.24 ± 17.89	< 0.001
	After intervention	51.79 ± 25.48	27.41 ± 11.84	
	*P*^b^	0.936	< 0.001	
Activity	Before intervention	41.58 ± 17.30	48.12 ± 13.02	< 0.001
	After intervention	49.05 ± 23.11	35.47 ± 13.83	
	*P*^b^	0.011	0.002	
Impact	Before intervention	29.42 ± 14.27	24.18 ± 15.17	0.003
	After intervention	32.42 ± 19.62	15.41 ± 13.19	
	*P*^b^	0.274	0.006	

*Abbreviations*: *6MWT* six minutes walking test, *FEV1* forced expiratory volume in one second, *FEV1%pred* percentage of predicted values of forced expiratory volume in one second, *FVC* forced vital capacity, *PT* peak torque, *BW* body weight, *TW* total work, *ER* endurance ratio, *SGRQ* St. George's Respiratory Questionnaire, *PQE* pulmonary-based Qigong exercise

-Values expressed as means and standard deviations

*P*^a^ values indicate comparison between groups by using the analysis of covariance, with the baseline values as the covariates

*P*^b^ values indicate comparison within groups by using paired *t*-test

#### Secondary efficacy outcome

##### Lung function test

Inter-group comparison showed no significant difference in FVC, FEV1% pred, FVC% pred, and FEV1/FVC% between PQE group and control group (*P* > 0.05, for each; Table 2), while there was a significant difference in FEV1 (*P* = 0.044; Table 2). When performing intra-group comparisons of PQE group and intra-group comparisons of control group, it was found that no statistically significant difference in FEV1, FVC, FEV1% pred, FVC% pred, and FEV1/FVC% (*P* > 0.05, for each; Table 2).

#### Skeletal muscle strength

##### Elbow isokinetic strength test

Inter-group comparison demonstrated no significant difference in PT, PT/BW, TW of extensor and flexor muscle (*P* > 0.05, for each; Table 2), and ER of flexor muscle (*P* = 0.081; Table 2), while there was a significant difference in ER of extensor muscle (*P* = 0.001; Table 2).

Intra-group comparison of control group revealed no significant difference in PT, PT/BW, TW and ER of extensor and flexor muscle (*P* > 0.05, for each; Table 2). Furthermore, the analysis of intra-group comparison in PQE group showed a significant difference in PT/BW of extensor and flexor muscle, and TW of extensor muscle (*P* < 0.05, for each; Table 2), however, there was no significant difference in PT and ER of extensor and flexor muscle, and TW of flexor muscle (*P* > 0.05, for each; Table 2).

##### Knee isokinetic strength test

Intergroup comparison showed that the improvement in PT, PT/BW, and TW of extensor and flexor muscle in the PQE group was significantly different compared with that in the control group (*P* < 0.05, for each; Table 2), however, no significant difference was observed in ER of extensor and flexor muscle (*P* > 0.05, for each; Table 2).

Intragroup comparison of control group demonstrated no statistically significant differences in PT and PT/BW of extensor and flexor muscle, TW and ER of extensor muscle (*P* > 0.05, for each; Table 2), however, there was a significant difference in TW and ER of flexor muscle (*P* < 0.05, for each; Table 2). In addition, the analysis of intragroup comparison in PQE group revealed a significant difference in PT of extensor muscle, and PT/BW and TW of extensor and flexor muscle (*P* < 0.05, for each; Table 2), and no significant difference in PT of flexor muscle, and ER of extensor and flexor muscle (*P* > 0.05, for each; Table 2).

##### Quality of life

Inter-group comparison demonstrated that the improvement in total and item score of SGRQ in the PQE group was statistically significant compared with that in the control group (*P* < 0.05, for each; Table 2).

Intra-group comparison of control group revealed no significant difference in total score, symptom score, and impact score (*P* > 0.05, for each; Table 2), while there was significant difference was observed in activity score (*P* = 0.011; Table 2). Additionally, intra-group comparison of PQE group showed the total and item score of SGRQ were decreased significantly (*P* < 0.05, for each; Table 2).

##### Dyspnea

In Table 3, intergroup comparisons showed no statistically significant differences in mMRC score (*P* = 0.082). Meanwhile, intragroup comparison of control group and PQE group demonstrated no statistically significant differences in mMRC score (*P* > 0.05, for each; Table 3).

**Table 3:** Comparison of mMRC in treatment groups before and after intervention

	**Control group (*n* = 19)**			**PQE group (*n* = 18)**			
	**Before intervention**	**After intervention**	***P***^**b**^	**Before intervention**	**After intervention**	***P***^**b**^	***P***^**a**^
Dyspnea							
mMRC			0.947			0.083	0.082
Grade 0	1 (5%)	1 (5%)		2 (11%)	2 (11%)		
Grade 1	3 (16%)	3 (16%)		3 (17%)	9 (50%)		
Grade 2	11 (58%)	9 (47%)		12 (67%)	5 (28%)		
Grade 3	4(21%)	6 (32%)		1 (5%)	2 (11%)		
Grade 4	0 (0%)	0 (0%)		0 (0%)	0 (0%)		

*Abbreviation*: *mMRC* Modified Medical Research Council, *PQE* pulmonary-based Qigong exercise

- Values are presented as means and standard deviations or n (%)

*P*^a^ values indicate inter-group comparison by using Fisher’s exact test

*P*^b^ values indicate intra-group comparison by using Fisher’s exact test

## Discussion

This study is an assessor-blinded, parallel, superiority, randomized clinical trial that evaluated the rehabilitative efficacy of PQE intervention for COPD-stable patients. The results may support our hypothesis that 3 months PQE intervention improves exercise capacity, skeletal muscle strength, and quality of life of COPD-stable patients and encourage the clinical use of PQE intervention as a safe and effective PR method in the family and community rehabilitation of COPD-stable patients.

TCE is a slow, gentle, self-healing aerobic exercise, which includes multi-component characteristics of isometric contraction, deep diaphragmatic breathing stretching muscle, and relaxation, with the main purpose of strengthening the body. Several clinical studies In recent years, have shown that TCE is suitable for elderly COPD patients and has positive rehabilitation effects [7]. As a modified TCE, the PQE reorganizes elements from Liu Zijue, Wu Qinxi, Ba Duanjin, and Yi Jinjing, and it has been developed from the basic theory of traditional Chinese medicine and aims at providing more targeted and practical exercise prescriptions for COPD-stable patients' functional recovery, as compared to a single type of TCE. Liu et al. [11] found that rearranged PQE significantly improved exercise capacity and activity of life, however, the effects on skeletal muscle strength, lung function, and physical activity are unclear. So, this trial aims to further evaluate the effects of PQE in COPD-stable patients using multidimensional outcomes.

Compared to conventional exercise training, TCE is easy to perform at home without any equipment or space limitations. Li et al. [20] proved that home-based Liu Zijue could improve lung function (FEV1), exercise capacity (6MWT), 30-s sit-to-stand test, and quality of life (SGRQ), furthermore, previous studies have also shown that 6 months of home-based Ba Duanjin and home-based Yi Jinjing significantly improved exercise capacity and quality of life [14, 21]. In brief, TCE can play a positive role in the long-term regular home-based rehabilitation of COPD-stable patients. Compared to the previous home-based TCE, this present study provides an easier-to-learn, more targeted, more practical, and effective home-based PQE intervention for COPD-stable patients, and the PQE intervention video, exercise record brochure registered by participants themselves, and encouragement and instruction from physiotherapists. In addition, the completion rate of 84% after 3 months of intervention in this trial is higher than 65% in a previous study that adopted the Ba Dunjin intervention in COPD-stable patients [14]. The high completion rates in this trial suggest that home-based PQE intervention is more accepted by COPD-stable patients based on practical clinical situations. In addition, we speculate that the age of the participants may have contributed to the attrition. Elderly COPD-stable patients may have difficulties with adhering to the PQE training for the full 3 months due to unstable disease states. Significantly, systemic and comprehensive methods should be conducted to determine the possible factors influencing attrition rates when PQE is used as an intervention.

Low to moderate-intensity aerobic training is effective in improving 6MWT and walking ability in COPD-stable patients [22, 23]. In this study, the PQE intervention, as low-moderate intensity aerobic training, showed a significant improvement in the exercise capacity of COPD-stable patients, as demonstrated by a mean increase of 34.52 m in 6MWT compared to pre-intervention. Noteworthy, Xiao et al. [24] obtained only a mean increase of 20.5 m in 6MWT after a 6 month of home-based Liu Zijue intervention for patients with COPD. It suggested that PQE might be more beneficial to improve 6MWT than a single TCE. Skeletal muscle dysfunction (SMD) is an independent risk factor for predicting mortality in patients with stable COPD [25] and is closely associated with decreased physical activity, exercise capacity, and quality of life [26]. Previous studies using TCE interventions for COPD-stable patients have rarely focused on SMD, in this trial, we used an isokinetic muscle strength test to objectively assess SMD in COPD-stable patients. Consequently, the study results showed that compared with the control group, 3 months of PQE intervention had a more significant advantage in improving the strength and endurance of the flexors and extensors of in COPD-stable patients, as demonstrated by significant improvements in the PT, PT/BW and TW of extensor muscle and flexor muscle of knee, and the ER of extensor muscle elbow. It is consistent with previous studies that found significant improvement in the isokinetic muscle strength test of the elbow in COPD-stable patients (age: 51 ± 10 years, FEV1%pred: 76 ± 23) after 12 weeks of endurance combined with resistance exercise (exercise intensity: 70% of maximum movement capacity), but with no significant improvement in elbow strength [27]. We speculated that the possible reason for the improvement of SMD by PQE intervention was that the prescribed pulmonary exercise includes more isometric contractions and slow isotonic contractions at different angles in the lower extremities, which could effectively stimulate the skeletal muscles of the lower extremities in COPD-stable patients. Furthermore, after intervention with PQE, both total and item scores of SGRQ decreased significantly and exceeded the minimum clinically important difference (MCID) of 4 points for the quality of life, as demonstrated by Jones [28], and the positive intervention effect of PQE on COPD-stable patients' quality of life was consistent with the intervention effect of TCE in previous studies [14, 21, 24].

TCE has been shown to significantly improve lung function in patients with stable COPD [7], surprisingly, 3 months of PQE intervention had no significant effect on lung function (except for the outcome of FEV1) in the present study, and the MCID (FEV1: 100 ml, FVC: 100 ml) has not been reached [29, 30]. We speculated that the possible reasons for no significant effect on the lung function test were that the participants included in this trial had inherently good levels of lung function (FEV1%pred: 59.18 ± 4.34), with less room for improvement, and that the 3 months intervention duration was short and had not yet reached the threshold for significant improvement. For the outcome of dyspnea, one study using the Borg scale to assess COPD patients after 3 months of Tai Chi intervention did not show a significant improvement in dyspnea, which was the same as the results of this study [31]. We speculated that this trial with a small sample size and included COPD patients under stable drug control, resulting in no significant difference in dyspnea. Thus, further studies are needed to show whether PQE intervention can have benefits on lung function and dyspnea.

## Limitations

Some limitations of this study should be acknowledged. First, there was no follow-up, so it is difficult to assess the effect of PQE intervention in improving the sustained rehabilitation of PR. Second, the sample size was relatively small, which might make it difficult to achieve significant differences in partial outcomes. Third, the sample of this trial was from a single source (single-center trial) and included a small number of women, so there might be regional differences and it was not possible to determine whether there were gender differences in the effects of the intervention. Future studies should improve these deficiencies to provide more definitive results.

## Conclusion

The findings of our study suggest that compared with the usual care provided, the proposed program of 3 months of PQE intervention has significant improvement in exercise capacity, skeletal muscle strength, and quality of life of COPD-stable patients. Furthermore, the PQE can be used as an alternative exercise program for COPD-stable patients in family and community rehabilitation. Further studies are needed to confirm the effects of this program and explore the optimal ways of clinical promotion.

### Supplementary Information

**Additional file 1. **

## Data Availability

We do not have a link to the data, and the data is in an SPSS file. If necessary, the datasets used and analyzed in the current study are available on request from the corresponding author upon reasonable request.

## Abbreviations

BW: Body weight
CONSORT: Consolidated Standards of Reporting Trial
COPD: Chronic obstructive pulmonary disease
ER: Endurance ratio
FEV1: Forced expiratory volume in one second
FEV1%pred: Percentage of predicted values of forced expiratory volume in one second
FVC: Forced vital capacity
MCID: Minimum clinically important difference
mMRC: Modified Medical Research Council
PQE: Pulmonary-based Qigong exercise
PR: Pulmonary rehabilitation
PT: Peak torque
SGRQ: St. George's Respiratory Questionnaire
SMD: Skeletal muscle dysfunction
SPSS: Statistical package for the social sciences
TCE: Traditional Chinese exercise
6MWT: Six minutes walking test
TW: Total work

## References

[1] Stolz D, Mkorombindo T, Schumann DM (2022). Towards the elimination of chronic obstructive pulmonary disease: a Lancet Commission. Lancet.

[2] Global initiative for chronic obstructive lung disease. Global strategy for the diagnosis, management, and prevention of chronic obstructive pulmonary disease (2023 REPORT). 2023. https://goldcopd.org/2023-gold-report-2/. Accessed 14 Nov 2022.

[3] Cavaillès A, Brinchault-Rabin G, Dixmier A (2013). Comorbidities of COPD. Eur Respir Rev.

[4] Donaldson AV, Maddocks M, Martolini D (2012). Muscle function in COPD: a complex interplay. Int J Chron Obstruct Pulmon Dis.

[5] Zeng Y, Jiang F, Chen Y (2018). Exercise assessments and trainings of pulmonary rehabilitation in COPD: a literature review. Int J Chron Obstruct Pulmon Dis.

[6] Siu PM, Yu AP, Chin EC (2021). Effects of Tai Chi or conventional exercise on central obesity in middle-aged and older adults: a three-group randomized controlled trial. Ann Intern Med.

[7] Luo X, Zhang J, Castelberg R (2016). The effects of traditional Chinese exercise in patients with chronic obstructive pulmonary disease: a meta-analysis. PLoS One..

[8] Ding M, Zhang W, Li KJ (2014). Effectiveness of t'ai chi and qigong on chronic obstructive pulmonary disease: a systematic review and meta-analysis. J Altern Complement Med.

[9] Dong XS, Wang XY, Jia NX (2021). A comparison between Qigong exercise and cycle ergometer exercise for the rehabilitation of chronic obstructive pulmonary disease: A pilot randomized controlled trial (CONSORT). Medicine (Baltimore).

[10] Liu X, Li P, Xiao L (2019). Effects of home-based prescribed pulmonary exercise by patients with chronic obstructive pulmonary disease: study protocol for a randomized controlled trial. Trials.

[11] Liu XD, Jin HZ, Ng HP, Gu YH, Wu YC, Lu G (2012). Therapeutic effects of Qigong in patients with COPD: a randomized controlled trial. Hong Kong J Occup Ther.

[12] Schulz KF, Altman DG, Moher D; CONSORT Group. CONSORT (2010). Statement: updated guidelines for reporting parallel group randomised trials. BMC Med.

[13] Global Initiative for Chronic Obstructive Lung Disease (GOLD). Global Strategy for the Diagnosis, Management and Prevention of Chronic Obstructive Pulmonary Disease (2017 Report). 2017. https://goldcopd.org/archived-reports/. Accessed 16 Nov 2016.

[14] Ng BH, Tsang HW, Jones AY, So CT, Mok TY (2011). Functional and psychosocial effects of health qigong in patients with COPD: a randomized controlled trial. J Altern Complement Med.

[15] Borg GA (1982). Psychophysical bases of perceived exertion. Med Sci Sports Exerc.

[16] ATS Committee on Proficiency Standards for Clinical Pulmonary Function Laboratories (2002). ATS statement: guidelines for the six-minute walk test.. Am J Respir Crit Care Med.

[17] Maffiuletti NA, Bizzini M, Desbrosses K, Babault N, Munzinger U (2007). Reliability of knee extension and flexion measurements using the Con-Trex isokinetic dynamometer. Clin Physiol Funct Imaging.

[18] Global Initiative for Chronic Obstructive Lung Disease (GOLD). Global Strategy for the Diagnosis, Management and Prevention of Chronic Obstructive Pulmonary Disease (2018 Report). 2018. https://goldcopd.org/archived-reports/. Accessed 15 Nov 2017.

[19] Sciriha A, Lungaro-Mifsud S, Scerri J, Magro R, Camilleri L, Montefort S (2017). Health status of COPD patients undergoing pulmonary rehabilitation: A comparative responsiveness of the CAT and SGRQ. Chron Respir Dis.

[20] Li P, Liu J, Lu Y, Liu X, Wang Z, Wu W (2018). Effects of long-term home-based Liuzijue exercise combined with clinical guidance in elderly patients with chronic obstructive pulmonary disease. Clin Interv Aging.

[21] Zhang M, Xv G, Luo C, Meng D, Ji Y (2016). Qigong Yi Jinjing Promotes Pulmonary Function, Physical Activity, Quality of Life and Emotion Regulation Self-Efficacy in Patients with Chronic Obstructive Pulmonary Disease: A Pilot Study. J Altern Complement Med.

[22] Chan AW, Lee A, Suen LK, Tam WW (2011). Tai chi Qigong improves lung functions and activity tolerance in COPD clients: a single blind, randomized controlled trial. Complement Ther Med.

[23] Vogiatzis I, Terzis G, Nanas S (2005). Skeletal muscle adaptations to interval training in patients with advanced COPD. Chest.

[24] Xiao CM, Zhuang YC (2015). Efficacy of Liuzijue Qigong in Individuals with Chronic Obstructive Pulmonary Disease in Remission. J Am Geriatr Soc.

[25] Passey SL, Hansen MJ, Bozinovski S, McDonald CF, Holland AE, Vlahos R (2016). Emerging therapies for the treatment of skeletal muscle wasting in chronic obstructive pulmonary disease. Pharmacol Ther.

[26] Sue DY (2003). Peripheral muscle dysfunction in patients with COPD: comparing apples to apples?. Chest.

[27] Clark CJ, Cochrane LM, Mackay E, Paton B (2000). Skeletal muscle strength and endurance in patients with mild COPD and the effects of weight training. Eur Respir J.

[28] Jones PW (2005). St. Georges Respiratory Questionnaire: MCID. COPD.

[29] Cazzola M, MacNee W, Martinez FJ (2008). Outcomes for COPD pharmacological trials: from lung function to biomarkers. Eur Respir J.

[30] Engel RM, Wearing J, Gonski P, Vemulpad S (2017). The effect of combining manual therapy with exercise for mild chronic obstructive pulmonary disease: study protocol for a randomised controlled trial. Trials.

[31] Chan AW, Lee A, Lee DT (2013). The sustaining effects of Tai chi Qigong on physiological health for COPD patients: a randomized controlled trial. Complement Ther Med.

