# Supplementary material for: Effects of pulmonary-based Qigong exercise in stable patients with chronic obstructive pulmonary disease: a randomized controlled trial
Source: BMC Complement Med Ther. 2023 Nov 20;23:418. doi: 10.1186/s12906-023-04238-8 (PMC10662926; doi:10.1186/s12906-023-04238-8)
Supplement: Supplementary file 1 — Additional file 1. [file 12906_2023_4238_MOESM1_ESM.docx]

**Additional file 1**

**Scheme of pulmonary-based Qigong exercise**

**Brief introduction**

Pulmonary-based Qigong exercise (PQE), following the basic theory of traditional Chinese medicine, combines and reorganizes elements from Liu Zijue, Wu Qinxi, Ba Duanjin, and Yi Jinjing to compile a new intervention of prescribed pulmonary exercise for COPD rehabilitation. The PQE intervention is more targeted and practical for COPD patients' functional recovery, which was developed by Xiao-Dan Liu (School of Rehabilitation Science, Shanghai University of Traditional Chinese Medicine, Shanghai, China) based on the disease characteristics of COPD. Previous research results of our research team show that the consistent practice of PQE has good improvement effects on chronic respiratory diseases, enhancing lung function, exercise capacity and quality of life, and relieving anxiety and depression in patients.

**Principle of exercise**

The first principle of PQE is safety. Patients who are at risk of falling should not train. It is recommended that the initial training be supervised by medical staff (or roommates), paying attention to the patient's subjective feelings (fatigue) and indicators such as heart rate, blood pressure and blood oxygen level during and after training; pursuing natural breathing, not forcing, not breathing hard, and prohibiting breath holding; progressively, the amplitude, number and duration of breathing and body movements can be gradually increased as needed, not pursuing one step; discomfort during training (such as induced cough, chest tightness, headache, dizziness, joint pain, etc.) should be promptly terminated, any discomfort during training (such as induced cough, chest tightness, headache, dizziness, joint pain, etc.) should be terminated in time; according to the individual's needs, some of the movements suitable for the patient can be selected for training.

**Scheme of exercise**

The program consists of nine characteristics: (1) Rise-up position, (2) “hu” sounding, (3) “si” sounding, (4) Pushing up the sky to regulate the triple warmer, (5) Drawing a bow to shoot a vulture, (6) Crane spread its wings, (7) Crane exercise of the crane flies, (8) Cross-armed iron staff, and (9) Restore position. Each time about 13-15 minutes. Train once in the morning and once in the afternoon.

**Prepared**

Stand naturally, feet apart, shoulder-width apart, knees slightly bent, head straight, neck straight, eyes front and bottom, arms hanging naturally at the sides of the body, breathing naturally, with a smile on your face and your whole body relaxed.

**First characteristic: Rise-up position**

Bend elbows, two palms ten fingers opposite each other, palms up, slowly up to the front of the chest, about the same height as the two breasts (inhale).

Both palms turn inward, palms downward, slowly press down to the navel. Slightly bend the knees and squat, the body sits back, while the two palms rotate inward, slowly move forward to the two arms into a circle (exhale). Rotate both palms outward, palms inward, rise (inhale), and slowly close both arms to the navel. Cross your tiger mouth and hold each other, rest for a while, breathe naturally and look at the front and bottom.

**Second characteristic: “hu” sounding**

Squat down with slightly bent knees, spit out the word "hu", while holding both palms outward (exhale, spit out the word "hu") until both arms are round. Then get up, both palms together (inhale), rest for one breath; then brace outside (exhale, spit "hu"), and so on for 6 times.

**Third characteristic: “si” sounding**

Following the above exercise, the two knees slowly straighten, while the two palms fall naturally, palms up, ten fingers opposite. Two palms slowly up to the chest, about the same height as the two breasts (inhale). Both elbows drop, clip the ribs, both hands stand in front of the shoulders, palms opposite each other, fingertips up. The two shoulder blades close to the spine, expand the shoulders and chest, hide the head and shrink the neck, look at the upper front (exhale - inhale, one breath). Squat down with your knees slightly bent, spit out the word fretting, while loosening your shoulders and stretching your neck, slowly push both palms forward and gradually turn them into a palm with the palms shining forward, looking forward (exhale, spit out the word "si"). Rotate the palms outward and turn the wrists until the palms are facing inward. Slowly straighten both knees, bend the elbows at the same time, and slowly close both palms to about 10 cm in front of the chest (inhale). Then drop your elbows, pinch your ribs, stand up your palms, expand your shoulders and chest, hide your head and shrink your neck, push your palms and spit out "si" (exhale), and repeat this exercise six times.

**Fourth characteristic: Pushing up the sky to regulate the triple warmer**

Following the above exercise, the two palms fall naturally, palm up, ten fingers opposite. Two palms five fingers apart, crossed in front of the abdomen, palm up, eyes forward, natural breathing. Rise up, two palms up in front of the chest (inhale), then two arms rotate upward, palms up, look up at the two palms. The two palms continue to hold up, elbow joints straight, while the jaws inward, the action stops, look ahead (exhale - inhale - exhale - inhale). Two knees squat, two arms respectively to the two sides of the drop (exhale), two palms cupped in front of the abdomen, palms up, eyes front. One up and one down for once, repeat the action 6 times.

**Fifth characteristic: Drawing a bow to shoot a vulture**

Following the above exercise, shift the center of gravity to the right, stand with the left foot open to the left, knees straighten slowly, cross the two palms upward in front of the chest, the left palm outside, look ahead (inhale). The right palm flexes its fingers and pulls to the right to the front of the shoulder, the left palm becomes the eight-letter palm, the left arm rotates inward and pushes out to the left, the same height as the shoulder, while both legs bend the knees into a horse stance, the action stops slightly, look at the left front (exhale). Weight right, both hands become natural palm, right hand arc to the right, the same height as the shoulder, palm oblique forward (inhalation), the center of gravity continue to move right, the left foot recovery into a parallel stance (exhale), while the two palms cupped in front of the abdomen, palm up, eyes front. The right movement is the same as the left, but the left and right opposite, left and right open bow like shooting eagle, a left and a right for a time, a total of 3 times.

**Sixth characteristic: Crane spread its wings**

Keep your feet apart, shoulder-width apart. When the two hands are raised, the shoulders are shrugged and the neck is contracted, the coccyx is upturned and the hands are horizontal (inhale). Relax the body on time, shift the center of gravity to the right and then extend the left leg backward to spread up (exhale), fold the two hands in front of the abdomen and lift them up to the top of the head, with the palms horizontal (inhale). Lean forward slightly, press the two hands down to the front of the abdomen and then backward to the back of the human herringbone separation (exhale). Both knees straight, keep the body stable. One left and one right for one time, a total of 3 times.

**Seventh characteristic: Crane exercise of the crane flies**

Following the above exercise, both hands together in front of the abdomen, side planks, lift the leg independently (inhale) and drop the standing leg (exhale). Then lift the leg up (inhale) and drop (exhale). The wrists are slightly higher than the shoulders in the side planks, the palms face each other in the drop, and the backs of the hands face each other in the upward lift, forming an upward flare. (When raising the knee with the standing leg, the supporting leg is straight. When descending, the supporting leg is then bent and the toes point to the ground, and then the knees are raised.) One left and one right for one time, 3 times in total.

**Eighth characteristic: Cross-armed iron staff**

Following the above exercise, natural breathing, two palms from the chest to the side of the body flat open, palms facing up (inhalation), into a double arm; palms turned down (exhalation), while the two feet heel up, toes on the ground, two eyes staring flat; heart flat air together. Style set for about 10-30 seconds, natural breathing.

**Ninth characteristic: Restore position**

Spread your feet apart, shoulder-width apart. Raise both hands sideways and upward, with inhalation. The body drops in front, with exhalation. Ideas along with the two hands, such as holding the air up to the top of the head when lifting, when falling outside the guide, the body is relaxed, the idea of going down. Both hands in front of the abdomen, arc together, tiger mouth crossed, folded in front of the abdomen, close your eyes and meditate, adjust your breathing.
